# Synthetic glycopeptides and glycoproteins with applications in biological research

**DOI:** 10.3762/bjoc.8.90

**Published:** 2012-05-30

**Authors:** Ulrika Westerlind

**Affiliations:** 1 Gesellschaft zur Förderung der Analytischen Wissenschaften e.V., ISAS – Leibniz Institute for Analytical Sciences, Otto-Hahn-Str. 6b, D-44227 Dortmund, Germany, Tel: (+49)231-1392 4215, Fax: (+49)231-1392 4850

**Keywords:** glycopeptide binding, glycopeptides, glycoprotein synthesis, solid-phase peptide synthesis, synthetic vaccines

## Abstract

Over the past few years, synthetic methods for the preparation of complex glycopeptides have been drastically improved. The need for homogenous glycopeptides and glycoproteins with defined chemical structures to study diverse biological phenomena further enhances the development of methodologies. Selected recent advances in synthesis and applications, in which glycopeptides or glycoproteins serve as tools for biological studies, are reviewed. The importance of specific antibodies directed to the glycan part, as well as the peptide backbone has been realized during the development of synthetic glycopeptide-based anti-tumor vaccines. The fine-tuning of native chemical ligation (NCL), expressed protein ligation (EPL), and chemoenzymatic glycosylation techniques have all together enabled the synthesis of functional glycoproteins. The synthesis of structurally defined, complex glycopeptides or glyco-clusters presented on natural peptide backbones, or mimics thereof, offer further possibilities to study protein-binding events.

## Introduction

The majority of human proteins are co- or post-translationally modified by mono- or oligosaccharides. The glycoprotein saccharides contribute physiochemical properties, influencing protein conformation or increasing stability against proteolytic activity. With their unique structural diversity and complexity, carbohydrates attached on proteins or lipids are involved in numerous cell-surface binding events, such as cell growth and differentiation, cell proliferation, cell adhesion, binding of pathogens, fertilization and immune responses [1–2]. Furthermore, glycans assist in intracellular protein folding and transport. Pathogenic processes, such as chronic inflammation, viral and bacterial infections, tumor growth and metastasis, and auto-immune disorders, all involve glycan cell–cell or cell–external-agent communication [3–5]. The availability of structurally defined glycopeptides and glycoproteins, which contain information about the glycan structure and glycosylation sites, is valuable for functional biological studies. Glycopeptides have, for instance, been applied to evaluate the role of conformational and proteolytic stability [6]. In other studies, synthetic glycopeptides have been employed in vaccines to induce specific immune responses or for the inhibition of protein-binding events [7–10]. This review is written with the intention to highlight a few recent reports describing the synthesis and application of synthetic glycopeptides and glycoproteins. For a more detailed description of this field of research, the reader is guided to other excellent reviews [6–18].

## Review

### Glycopeptide-based vaccines

Specific immune recognition, in which the glycan and the peptide backbone contribute to the binding epitope, is of particular interest for the development of safe immunotherapy and immunodiagnostics. Since the discovery, by Springer et al., that glycoproteins on the outer cell membrane of epithelial tumor cells have an altered glycosylation consisting of the Thomsen-Friedenreich (T-) antigen and its precursor T_N_-antigen structure, the synthesis and evaluation of anti-tumor vaccines have been a topic of intense research [19]. During the past few years, the synthesis and evaluation of glycopeptide-based mucin anti-tumor vaccines have dominated this research area [7,20–24].

**Figure 1:**
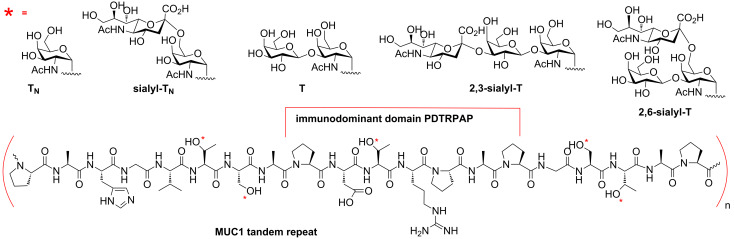
Tumor-associated glycosylation on MUC1 tandem repeat peptides.

Mucins are a class of extensively glycosylated proteins expressed on the surface of epithelial cells or secreted in mucus. Among them, mucin 1 (MUC1) is expressed on almost all epithelial tissues. Changes of the cell-surface protein glycosylation together with MUC1 protein overexpression, results in the formation of tumor-specific epitopes consisting of both the formed short saccharides, e.g., T_N_, T, sialyl-T_N_ and sialyl-T, and the mucin tandem repeat peptide region, which is exposed due to the aberrant glycosylation (Figure 1) [25–27]. A number of synthetic glycopeptide vaccines with the MUC1 tumor associated glycopeptide epitope as target have recently been prepared. Variation of glycan structure, number of glycans per repeat, and the sites for glycan attachment on the MUC1 peptide backbone were explored. For the induction of a strong and specific immune response, different immuno-stimulants were connected to the mucin glycopeptides. Among the immuno-stimulants, the Toll-like receptor 2 (TLR2) ligand, tripalmitoyl-(*S*)-glyceryl lipopeptide Pam_3_-Cys-Ser-(Lys)_4_ (Pam_3_CSK_4_) and carrier proteins, such as tetanus toxoid (T.Tox.), have been successfully applied in MUC1 anti-tumor vaccines [28–30]. T_H_-cell peptides included in two- or three-component vaccines and multivalent glycopeptide dendrimer vaccines have furthermore been evaluated [31–33].

Synthetic MUC1-tetanus toxoid conjugate vaccines have proven to be particularly interesting. Tetanus toxoid vaccines conjugated to other antigens have been administered to humans. During immunization in mice, highly specific and strong immune responses were induced in a number of cases [28–29,34]. Furthermore, several antibodies induced by the vaccines showed specific recognition of tumor cells.

**Scheme 1:**
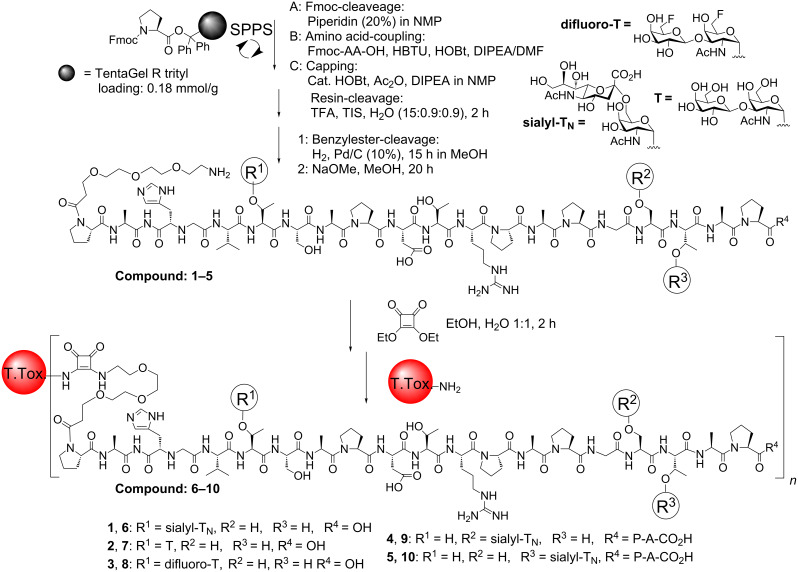
Synthesis of MUC1 tetanus toxoid protein conjugate vaccines.

The MUC1 tandem repeat glycopeptides were synthesized on solid-phase, according to the Fmoc strategy, by using Fmoc protected amino acids and Fmoc glycosyl amino acid building blocks in a stepwise fashion. After cleavage from resin and removal of the protecting groups the MUC1 glycopeptides (**1**–**5**) could be conjugated through diethyl squarate to the tetanus toxoid carrier protein (Scheme 1). Immunological evaluation of MUC1 glycopeptide vaccines containing a sialyl-T_N_, a T-antigen, or a difluoro-T-antigen on different positions in the tandem repeat (**6**–**10**), showed that high antibody titers were induced in almost all of the immunized mice. Evaluation of the generated antibodies provided evidence that specific immune responses were elicited towards the MUC1 antigens present in the vaccines. FACS analysis with serum antibodies induced by the MUC1 vaccines **7–10** showed that they all recognize MCF-7 breast-cancer cells. The serum from mice immunized with vaccine **9** was further evaluated through mammary carcinoma tissue-staining experiments. A gradual increase of the tissue staining was found, resulting from a strong binding to the advanced G3-phase tumor tissue. Taken together, these results show that a selective immune response discriminating between healthy and diseased tissue can be efficiently obtained [28–29,34].

**Scheme 2:**
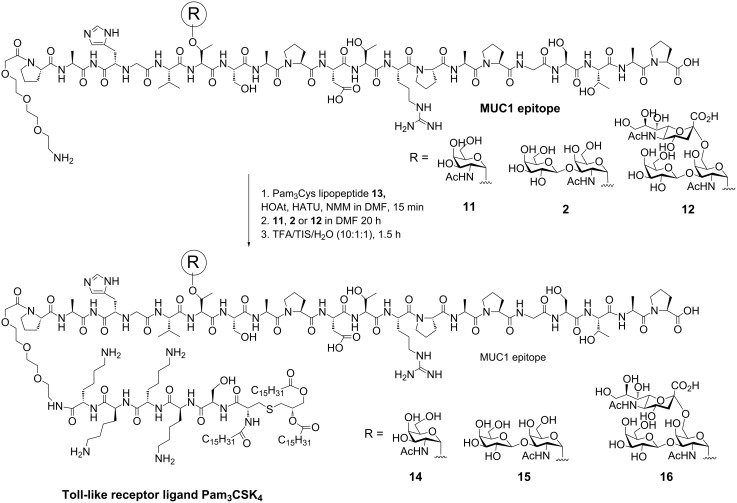
T_N_, T and sialyl-T MUC1 Pam_3_Cys two-component vaccines.

A number of two- or three-component vaccines containing a Toll-like receptor-2 (TLR2) ligand have, moreover, been synthesized and evaluated [35–38]. These self-adjuvanting vaccines avoid invoking an immune response to the immune carrier, a problem commonly seen upon employing protein conjugate vaccines. The synthesis of two-component vaccines with a Pam_3_Cys TLR2 ligand connected to T_N_, T and sialyl-T MUC1 glycopeptides was recently described (Scheme 2) [35]. The Pam_3_CSK_4_ lipopeptide fragment **13** was prepared by Fmoc solid-phase synthesis, including protected amino-acid side chains after resin cleavage. Subsequently, different T_N_, T and sialyl-T MUC1 glycopeptides (**2**, **11**, **12**) were prepared and fully deprotected. Peptide synthesis was followed by fragment condensation employing HATU and HOAt and after additional deprotection steps, resulting in the formation of the lipopeptide vaccine constructs **14–16**. Immunization of the vaccines in mice showed that a specific immune response was induced in all mice although not with the high antibody titers found upon employing MUC1 tetanus toxoid vaccines [35]. In a later study, two- and three-component vaccines were synthesized and compared by immunological evaluation. The three component vaccines, containing an extra tetanus toxoid T-cell peptide epitope, showed a stronger immune response when the MUC1 peptides were glycosylated, whereas the nonglycosylated two- and three-component vaccines did not show any difference in antibody titers [36]. Recently, a three-component vaccine consisting of a MUC1 T_N_-glycopeptide, a polio peptide T-cell epitope and the Pam_3_CSK_4_ lipopeptide immune-stimulant was prepared by liposome-mediated native chemical ligation. Mice immunized with the three-component vaccine were injected with breast-cancer tumor cells, and were found to be significantly more resistant to tumor growth compared to control mice [37]. Vaccines with multivalent MUC1 T- and T_N_-glycopeptides were efficiently conjugated through azide/alkyne click chemistry to the Pam_3_CSK_4_ lipopeptide immune-stimulant. The recently reported vaccines are currently under immunological investigation [38].

### Synthetic glycoproteins

**Scheme 3:**
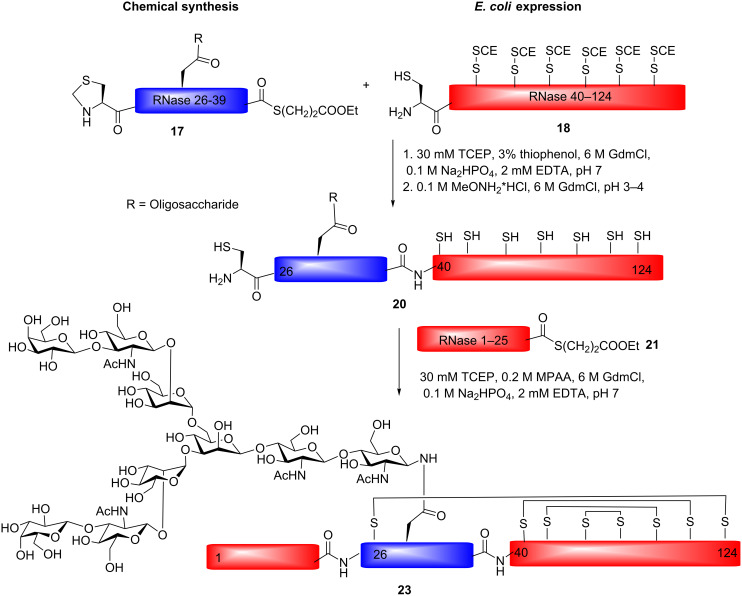
Preparation of RNase C by sequential NCL.

An important task for chemists is to make homogenous glycoproteins and complex glycopeptides available for biological research. Studies of glycosylation effects on protein conformation, stability and structure–activity relationships (SAR) are a few examples of the applications of synthetic glycoproteins. Since the discovery of native chemical ligation (NCL) by Kent and co-workers, numerous efforts have been made to prepare challenging protein targets [39–44]. In the NCL method, a native amide bond is formed by coupling of a C-terminal thioester with the N-terminal cysteine, followed by a S→N acyl shift. The development of a variant of NCL, namely expressed protein ligation (EPL), has further made it possible to efficiently prepare large proteins without the size limitations set by ordinary peptide synthesis [45]. Bertozzi and co-workers introduced the NCL and EPL techniques for glycoprotein synthesis by preparation of a number of GalNAc containing *O*-glycoproteins, such as the antimicrobial protein diptericin, the cytokine lymphotactin and the leukocyte adhesion molecule ligand GlyCAM-1 [46–48]. By repeated NCL couplings of mucin tandem repeats, MUC2 and MUC1 tandem repeat polypeptides have been prepared, and such polypeptides are useful for the development of anti-tumor vaccines [49–50]. The bovine ribonuclease C glycoprotein (RNase C) has been used as a model system for the preparation of homogenous *N*-glycoproteins, employing NCL and EPL for coupling between a *N*-glycopeptide and other peptide/protein fragments [51–53]. The glycoprotein contains eight cysteines locked up as disulfide bridges, which need to be correctly folded to obtain an active enzyme [54–56]. In one study, a complex type *N*-glycopeptide (fragment **17**) was prepared by standard Fmoc-SPPS followed by sequential NCL [51]. The *N*-glycopeptide fragment RNase 26–39 (**17**) was prepared with a thioester in the C-terminal and a thiazolidine protected cysteine at the N-terminus. The chemical ligation was performed by coupling of the *N*-glycopeptide thioester RNase 26–39 (**17**) and the expressed protein fragment 40–124 (**18**) containing a N-terminal cysteine, employing thiophenol and tris(2-carboxyethyl)phosphine (TCEP). The obtained RNase fragment 26–124 (**19**) was then treated with *N*-methoxyamine (0.2 M, pH 3–4, 4 h) to remove the protecting group on the N-terminal cysteine. Ligation of the RNase fragment 26–124 (**20**) to the thioester peptide fragment RNase 1-25 (**21**) was followed to give RNase fragment 1–124 (**22**). The formed RNase C protein was then folded by treatment with glutathione disulfide (GSSG) resulting in an active RNase C enzyme **(23)** (Scheme 3).

Recently, the 111-amino-acid long β-subunit of human follicle-stimulating hormone (hFSH) glycoprotein was prepared, containing two complex type *N*-glycans, modified with core fucose and terminal sialic acid glycan residues [57]. The FSH β-subunit was prepared by sequential native chemical ligation. Initially, a larger C-terminal fragment, (β-FSH 28–111) was obtained by ligation of two shorter synthetically prepared peptides followed by sequential coupling of two N-terminal glycopeptide fragments (β-FSH 1–19 and 20–27) employing standard NCL conditions. Binding of the hFSH glycoprotein to its receptor stimulates the maturation of follicles and the production of estrogene in females, and maintains spermatogenesis in males [58]. Recombinant FSH, which is heterogeneously glycosylated, is currently used in the clinic for treatment of disorders associated with infertility [59]. Studies on mice have further indicated that FSHs reduce tumor growth [60]. The synthesis of different homogenous glycosylated FSH proteins to investigate SAR would therefore be highly desirable.

**Scheme 4:**
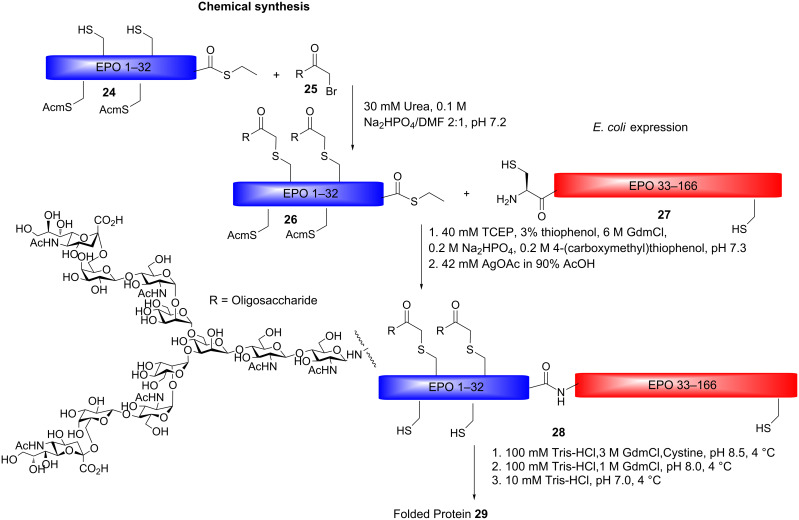
Preparation of an EPO analogue modified with complex type *N*-glycans at position 24 and 30.

Another clinically relevant glycoprotein prepared by NCL and EPL is erythropoietin (EPO), a red-blood-cell stimulant used for the treatment of renal anaemia [61]. EPO consists of 166 amino-acid residues with four glycosylation sites, one O-glycosylation site positioned at serine 126 and three N-glycosylation sites positioned at aspargines 24, 38 and 83. EPO has been found to contain multiple glycoforms and the efficacy of EPO is heavily dependent on the type and extent of glycosylation [62–63]. The synthesis of homogeneously glycosylated EPO for improvements in therapy is therefore desirable. Kajihara and co-workers have prepared a number of erythropoietin analogues by expressed protein ligation [64–65]. Complex-type *N*-glycans were attached through coupling to side-chain cysteine residues employing the haloacetamide method, forming a non-native linkage to the peptide backbone (Scheme 4). Due to *E. coli* expression of fragment 33–166, the natural sites for glycosylation 38, 83 and 126 could not be modified, and instead, glycosylations were incorporated at other unnatural positions. The synthetic EPO analogues modified with two or three complex type *N*-glycans, were evaluated in a cell proliferation assay showing that the analogues induced cell-proliferation in vitro. However, cell-proliferation activity in vivo was very weak for both analogues.

Danishefsky and co-workers prepared a number of erythropoietin glycopeptide fragments to improve the native chemical ligation methodology [14,66–68]. Recently the complete EPO protein was prepared by sequential NCL containing four glycosylation sites [69].

Although traditional NCL has resulted in advancements in the preparation of synthetic proteins, there is still a need for better methods that are independent of the relatively low abundance of the cysteine residue for a broad application of NCL in protein synthesis. To meet these needs, efforts are being made to improve the NCL ligation method, e.g., auxiliary based protocols, desulfurization methods or sugar-assisted chemical ligation (SAL) are all examples of expanded NCL techniques [70–77].

**Scheme 5:**
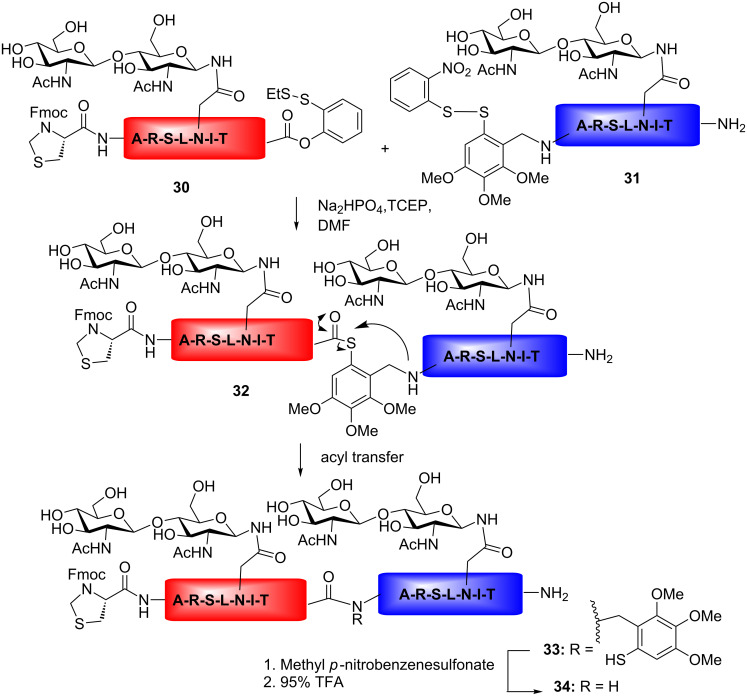
Auxiliary-assisted NCL of two glycopeptide fragments.

According to the auxiliary-based ligation strategy, a thiol-containing mimic replaces the N-terminal cysteine; the mimic group is then cleavable after ligation with the C-terminal thioester peptide fragment. The auxiliary-based coupling strategy employing the 4,5,6-trimethoxy-2-mercaptobenzoyl (Tmb) group is illustrated in Scheme 5. Two glycopeptide fragments are coupled together under reductive conditions. The *ortho*-thiophenolic moiety rearranges, forming a C-terminal thioester, followed by coupling to the N-terminal peptide fragment containing a thiol auxiliary. After coupling, the Tmb auxiliary could be removed by treatment with 95% trifluoroacetic acid (TFA). In spite of the strong-acid treatment required, the glycosidic bonds were reported to stay intact (Scheme 5) [70,78].

**Scheme 6:**
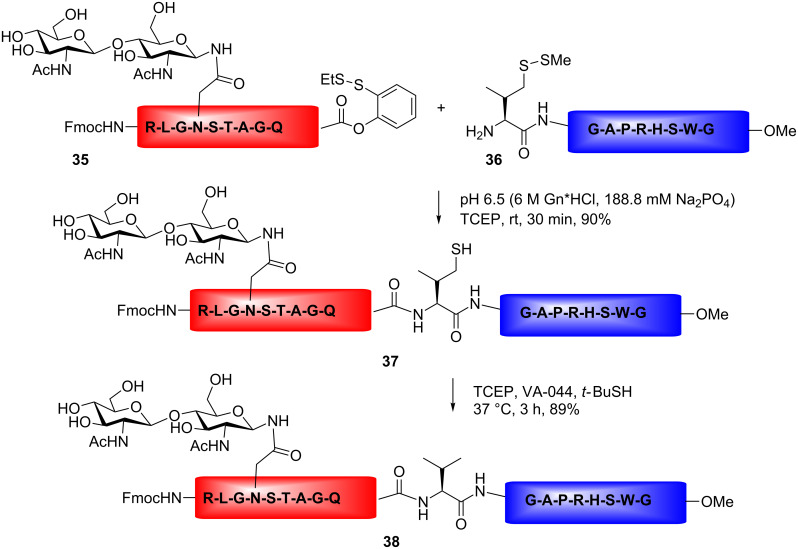
NCL of a glycopeptide fragment, generating valine by desulfurization at the ligation site.

Although applicable in glycopeptide ligation, the Tmb auxiliary strategy is of limited use in glycoprotein synthesis, due to the harsh conditions employed for auxiliary removal. Furthermore, due to steric hindrance by the auxiliary group, the reactivity of the amine group is reduced resulting in longer coupling times and lower yields. Therefore, the auxiliary method is mainly applied when the ligation site contains a glycine residue. To avoid these limitations, a ligation methodology has been developed with the thiol group linked to the amino-acid side-chain functional groups. After ligation the side-chain thiol could be removed by Raney nickel treatment. Employing the desulfurization methodology, thiol amino acids could be converted to alanine, valine, phenylalanine and threonine residues [71–76]. As an alternative to Raney nickel reduction, a radical-induced desulfurization method was recently reported [76]. An example of the desulfurization method is illustrated in Scheme 6. After the NCL-reaction, the γ-thiol valine at the ligation site could be converted to a valine by treatment with tris(2-carboxyethyl)phosphine (TCEP), *t*-BuSH, and the water-soluble radical initiator VA-044 [73]. The radical-induced desulfurization method has also been applied in the total synthesis of an EPO glycoprotein analogue [69].

**Scheme 7:**
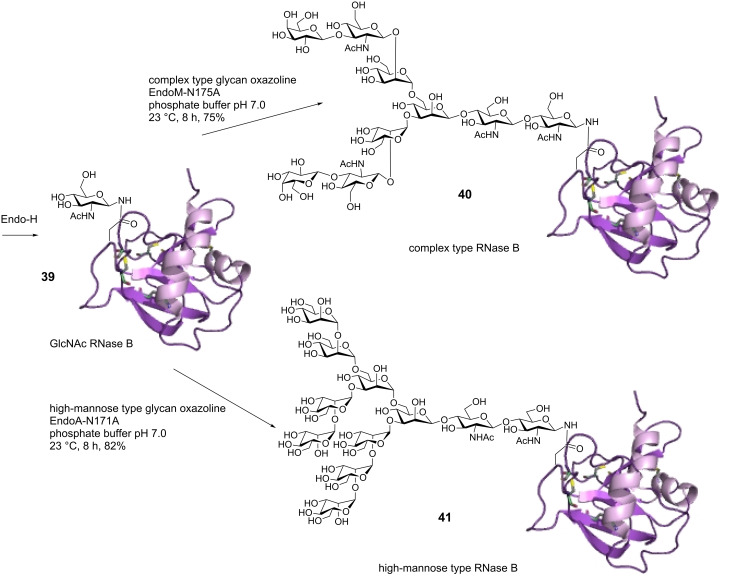
Synthesis of homogeneous glycoproteins by chemoenzymatic glycan remodeling.

Another promising path to prepare homogenous glycoproteins is represented by chemoenzymatic synthesis. Both glycosyltransferases and endoglycosidases have been applied in the synthesis or modulation of the glycan structure of glycopeptides and glycoproteins [79–80]. The chemoenzymatic strategy based on transglycosylation activity of endo-β-*N*-acetylglucosaminidase (ENGase) deserves particular attention. Two ENGases have commonly been applied in glycopeptide/glycoprotein synthesis, Endo-A specific for high-mannose glycans and Endo-M operating on both high-mannose and complex type *N*-glycans [81–83]. These enzymes can, in contrast to glycosyltransferases, by means of a one-step reaction attach large oligosaccharides to a GlcNAc polypeptide. Until recently, the application of the transglycosylation reaction was of limited use, due to competing enzyme hydrolysis of the formed glycopeptide/protein. By use of oxazoline transition-state analogue substrates, the rate for the transglycosylation reaction was increased and thereby the slower product hydrolysis reaction could be avoided [84–85]. A number of successful preparations of *N-*glycopeptides and *N-*glycoproteins have since then been successfully prepared by using oxazoline oligosaccharide donors; the synthesis of a HIV gp120 glycopeptide fragment, the HIV gp41 peptide, and the RNase B glycoprotein are a few examples [84,86–89]. The glycan remodeling approach is particularly interesting for glycoprotein synthesis. This method was employed in the synthesis of two RNase B glycoproteins modified with either high-mannose or complex type *N*-glycans [87]. The bovine RNase B glycoprotein **39** used in this study contained a mixture of high-mannose glycoforms; by treatment with endoglycosidase H (Endo-H), these glycans could be hydrolytically cleaved leaving the GlcNAc monosaccharide still attached to the protein. By treatment with a complex type glycan oxazoline donor and an Endo-M mutant or a high-mannose oxazoline donor and an Endo-A mutant, two different homogenous glycoproteins **40** and **41** were formed (Scheme 7) [87].

### Glycopeptide binding events

Carbohydrates on proteins and lipids play critical roles in cell–cell and cell–external-agent binding events. Efforts in the preparation of diverse carbohydrate structures immobilized in a microarray format have made carbohydrates accessible for numerous functional studies of protein-binding recognition [90–92]. In addition to the glycan structure, multivalency and orientation are important for the presentation of glycan ligands, and, in addition, the peptide backbone of the glycoprotein is sometimes a part of the binding recognition domain. Synthetic glycopeptides with natural multivalent presentation of glycan structures may function as tools for the investigation of protein-binding events. Applications of glycopeptides in a microarray format are desirable for such binding studies. Evaluation of antibody binding epitopes in vaccine and biomarker discovery is one recent example of glycopeptide microarrays [93–94].

Multivalency and orientation for glycan presentation have been shown to be important for the inhibition of microbe and lectin binding [95–105]. The synthesis of glycoclusters/dendrimers is an area of research aimed at tackling the increasing problems with bacterial multi-antibiotic resistance. Microbe adherence to the glycans on the tissue cell surface is essential for an infection to progress. As a consequence, mutations of the pathogen adhesion proteins, resulting in a loss of cell-surface binding recognition are not very likely to occur. Interference with the microbe binding events by employing an anti-adhesive strategy could therefore be very efficient. In several microbe and lectin binding studies, glycopeptide based glycoclusters/dendrimers were applied, employing linear peptide backbones, cyclic peptide scaffolds or multi-lysine scaffolds [106–118].

The pentavalent cholera toxin protein secreted by *Vibrio cholerae,* causes severe diarrhea and massive dehydration upon binding and entrance into the intestinal epithelial cells [119]. The AB_5_-type toxin consists of one toxic ADP-ribosyltransferase and five lectin subunits that bind to the gangloside GM1 ligands on the epithelial cell surface [120]. The cholera-toxin–GM1 complex is one of the most well characterized protein–carbohydrate interactions [95–99]. Development of inhibitors targeting the cholera-toxin protein–carbohydrate binding events is a novel strategy for disease prevention and therapy as well as for detection of the toxin in patient samples. Binding studies to GM1 ligands have clearly demonstrated the importance of multivalency in protein–carbohydrate binding recognition [95–99,120]. In one study, a 380,000 (47,000/sugar)-fold affinity enhancement and an IC_50_ of 50 pM was reported, by comparison of a GM1 monovalent derivative with the multivalent inhibitor [121]. The X-ray crystal structure of the cholera-toxin–GM1-ganglioside complex has shown that most of the protein contacts are given to the terminal galactose unit [122]. With this knowledge in hand, dendrimers based on readily available galactose monosaccharide ligands have been prepared and evaluated [123].

**Figure 2:**
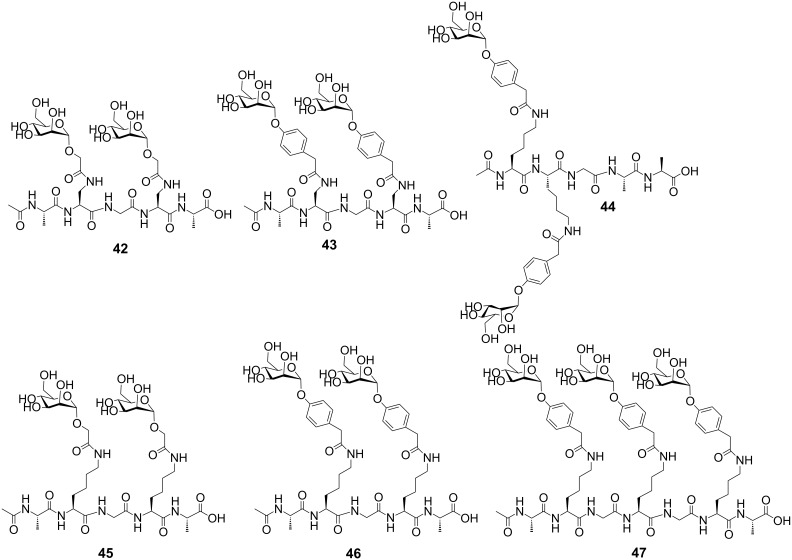
Di- and trivalent glycopeptide dendrimers evaluated for FimH inhibition.

The type 1 fimbriated *Echerichia coli* is a pathogen responsible for urinary tract infections with millions of cases every year [124]. The type 1 fimbriae have been identified to be a major contributor to these infections [125–126]. The FimH lectin on type 1 fimbriae is an attractive target for the inhibition of α-mannose-mediated cell adhesion [127–131]. Previous X-ray studies have proven that the FimH lectin has a monovalent binding site recognizing α-*D*-mannose [132–133]. In close proximity to the mannose-binding crevice, two tyrosine residues form the “tyrosine gate” [134]. By π–π stacking interactions with the aromatic tyrosine residues, monovalent α-mannose ligands containing hydrophobic aglycons, have shown increased binding affinities [128,135–136]. Employing multivalent ligands, the binding affinity to FimH could be further increased [137–139]. In one study, the FimH inhibition of mannosylated di- and tetravalent lysine core dendrimers resulted in 455- and 2000-fold increases relative to a monovalent mannose residue [114]. The observed multivalency effects are not fully understood. In a recent study, mannose di- and trivalent glycopeptides were evaluated for their inhibition of FimH binding [107]. The valency, conformational properties, and spatial arrangement of the attached mannose residues were evaluated. Glycopeptides containing an aromatic aglycon showed increased affinity to FimH due to interactions with the FimH tyrosine gate; this effect was more pronounced by the divalent glycopeptides. In accordance with other studies, it could be concluded that the distance between the mannoside ligands was important, showing stronger inhibition for the divalent glycopeptide with a larger spatial ligand distance (Figure 2) [140–142].

In another study, a combinatorial library of fucosyl-peptide dendrimers was synthesized and screened for binding to the fucose-specific lectin (LecB) from *Pseudomonas aeruginosa*, a pathogen causing severe infections in patients leading to chronic inflammation in the airways [106,143]. Previously, it was found that the LecB protein was important for biofilm formation [144–146]. In the screening for LecB inhibitors, one glycopeptide dendrimer, FD2 **49** (C-FucLysProLeu)_4_(LysPheLysIle)_2_LysHisIleNH_2_, showed particularly strong LecB inhibition (FD2 IC_50_ = 0.14 μM and L-fucose IC_50_ = 11 μM) [106]. The glycopeptide dendrimer was able to completely inhibit *P. aeruginosa* biofilm formation at a concentration of 50 μM and established biofilms from wild-type strain and clinical isolates could be completely dispersed. In a later study, analogues of FD2 **49** were prepared; in one of them the L-amino acids were replaced by D-amino acids (D-FD2, **51**) to avoid proteolytic cleavage of the peptide construct [147]. Interestingly, it was found that the D-FD2 **51** glycopeptide dendrimer showed a slightly weaker binding affinity to LecB, but the *P. aeruginosa* biofilm inhibitory properties remained, whereas D-FD2 **51**, in contrast to FD2 **49**, was completely resistant to proteolysis (Table 1) [147].

**Table 1:** Synthesis of LecB glycopeptide dendrimer ligands for biofilm inhibition.

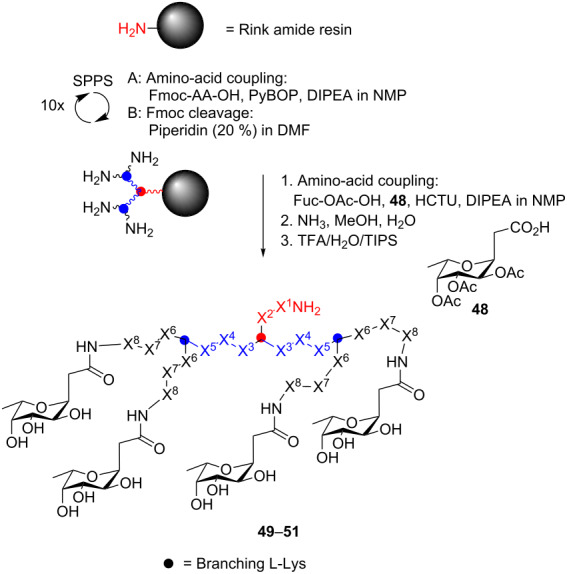								

	X^8^	X^7^	X^6^	X^5^	X^4^	X^3^	X^2^	X^1^

**FD2** (**49**)	Lys	Pro	Leu	Phe	Lys	Ile	His	Ile
Leu**-FD2** (**50**)	Lys	Pro	Leu	Phe	Lys	Leu	His	Leu
D**-FD2** (**51**)	D-Lys	D-Pro	D-Leu	D-Phe	D-Lys	D-Leu	D-His	D-Leu

Considering the favorable properties of the FD2 LecB inhibitor, an identical peptide dendrimer backbone was employed for the development of inhibitors of the galactose-specific *P. aeruginosa* lectin LecA [148]. By incorporation of hydrophobic groups to the galactose anomeric position, the affinity to LecA could be further enhanced. The most potent LecA inhibitor from these studies, GalAG2, showed a 4000-fold increase in hemagglutination inhibition activity and 875-fold increase in binding (*K*_d_) to LecA compared with D-galactose [148]. Similar to the LecB inhibitor FD2, complete inhibition of biofilm formation was observed.

## Conclusions

Recent developments of synthetic methodologies have enabled the preparation of complex glycopeptides and glycoproteins. The availability of structurally defined material has further made it possible to apply glycopeptides in biological studies. The development of glycopeptide anti-tumor vaccines and the synthesis of homogenous glycoproteins and glycopeptide dendrimers for the inhibition of microbe binding events are examples described in this review. The development of methods in the synthesis of homogenous glycoproteins may result in new therapeutic applications. Better understanding of protein glycosylation will help to identify new protein targets for immunotherapy. The synthesis and application of glycopeptide microarrays is an upcoming topic, which will offer multivalent presentation and fine-tuning in studies of specific protein-binding events.
